# Optical coherence tomography measurement of retinal layers above the peripapillary hyperreflective ovoid mass-like structure in true papilloedema and pseudo-papilloedema

**DOI:** 10.1038/s41433-026-04522-0

**Published:** 2026-05-21

**Authors:** Christopher Maximilian Behrens, J. Alexander Fraser, Sanja Cejvanovic, Elisabeth Wibroe, Judith Warner, Steffen Hamann

**Affiliations:** 1 Department of Ophthalmology, Copenhagen University Hospital - Rigshospitalet, Glostrup, Denmark; 2 Department of Clinical Neurological Sciences, Western University, London, ON Canada; 3 Department of Ophthalmology, Western University, London, ON Canada; 4 Moran Eye Center, University of Utah, Salt Lake City, Utah USA; 5 Department of Clinical Medicine, University of Copenhagen, Copenhagen, Denmark; 6 Rothschild Foundation Hospital, Paris, France

**Keywords:** Predictive markers, Retina

## Abstract

**Background:**

Discriminating between true papilloedema and pseudo-papilloedema is crucial, as true papilloedema can be both vision- and life-threatening.

**Objectives:**

To test a simple method for differentiating pseudo-papilloedema from true papilloedema in patients with peripapillary hyperreflective ovoid mass-like structures (PHOMS) using optical coherence tomography (OCT).

**Subjects:**

81 patients underwent OCT scanning of the optic nerve head (ONH) and peripapillary region following systematic guidelines. Patients were categorised into two groups: true papilloedema due to idiopathic intracranial hypertension (*n* = 34) and pseudo-papilloedema due to optic disc drusen (*n* = 40) or myopic tilted discs (*n* = 7).

**Methods:**

PHOMS dimensions and thickness of retinal sublayers above PHOMS were measured. The retinal thickness directly above the PHOMS and perpendicular to the internal limiting membrane (the PHOMS UP thickness) was measured.

**Results:**

While PHOMS were equal in height and width between groups, the PHOMS UP thickness was significantly larger in the true papilloedema group than in the pseudo-papilloedema group (378 µm ( ± 70 µm) vs 204 µm ( ± 47 µm), *P* < 0.001).

**Conclusions:**

In this selected cohort of patients with well-defined PHOMS, the significantly larger PHOMS UP thickness in the true papilloedema group supports the hypothesis that this condition involves both intra-axonal and interstitial fluid stasis, while pseudo-papilloedema only involves intra-axonal axoplasmic stasis. A PHOMS UP thickness greater than 300 µm may be suggestive of true papilloedema in this context. PHOMS UP thickness may serve as an additional, simple, rapid, OCT-metric to aid in differentiating pseudo-papilloedema from true papilloedema in patients with PHOMS.

## Introduction

A peripapillary hyperreflective ovoid mass-like structure (PHOMS) is a well-described marker of nonspecific axoplasmic stasis seen on optical coherence tomography (OCT) scans of the optic nerve head (ONH). Histopathologically, a PHOMS corresponds to distended axons bulging or herniating laterally into the peripapillary space, displacing the peripapillary retina upward due to its mass effect [1, 2].

PHOMS have recently been identified in up to 9% of 10–11-year-old children in a cohort of 1400 healthy Danish children without optic disc drusen (ODD) or papilloedema and are consistently associated with high myopia and tilted ONHs in both western and Asian children [3–5].

PHOMS occur in various ONH disorders and can be classified into ODD-associated (type 1), anomalous-disc-associated (type 2) and optic disc oedema-associated (type 3) PHOMS. Types 1 and 2 are signs of pseudo-papilloedema, while type 3—when due to elevated intracranial pressure—may indicate true papilledoema [1, 6, 7].

There is growing demand for simple, rapid, non-invasive tools for distinguishing pseudo-papilloedema—common in children and individuals with myopia—from true papilloedema. Accurate differentiation is crucial, as their underlying causes and management differ, the former being a common and benign finding and the latter being potentially life- and vision-threatening caused by underlying conditions such as intracranial brain tumour, hydrocephalus, cerebral venous sinus thrombosis or idiopathic intracranial hypertension (IIH) [7].

This study aimed to evaluate a simple, accessible method to differentiate papilloedema from pseudo-papilloedema using ONH OCT measurements. We theorise that in pseudo-papilloedema, only intracellular oedema from axoplasmic stasis is present, forming a PHOMS and the overlying retinal layers (termed the PHOMS UP layer) appear thickened solely due to the mass effect of the PHOMS. In contrast, true papilloedema involves both intracellular and extracellular (interstitial) fluid, leading to thickened PHOMS UP layers. The hypothesis was, therefore, that the PHOMS UP thickness is significantly greater in type 3 PHOMS with true papilloedema than in type 1 and 2 PHOMS with pseudo-papilloedema. If correct, the PHOMS UP measurement technique could serve as a simple, fast and widely available OCT-based tool aiding in differentiation.

## Subjects and methods

### Study type and study population

This was a cross-sectional, retrospective study of consecutive patients examined for papilloedema or pseudo-papilloedema from January 2016 to January 2025 in the Neuro-ophthalmology clinics of Rigshospitalet, Copenhagen, Denmark and Western University, London, Canada. Ethical approval was obtained from the research ethics committee of the capital region of Denmark and the research ethics board of Western University. The study adhered to the tenets of the Declaration of Helsinki. Inclusion criteria were: (1) radial or midline OCT scans with PHOMS confirmed by a neuro-ophthalmologist (SH or JAF) in association with (a) ODD, defined as hyporeflective, rounded ONH structures [6], (b) myopic tilted ONH, defined by oblique insertion of the optic canal plane into the BMO plane [8, 9]), or (c) IIH, diagnosed according to the 2013 revised diagnostic Friedman criteria [10], (3) age 18–45 years; and (4) signed consent. To ensure assessment of the PHOMS-related mass effect on the overlying retinal layers, we predefined thresholds of PHOMS prior to data extraction, so that only medium to large-sized PHOMS were included. Therefore, specific OCT-based inclusions were added, requiring (5) PHOMS involving ≥25% of the peripapillary circumference; (6) PHOMS cross-sectional height ≥300 µm on the central section and ≥150 µm in adjacent sections. Exclusion criteria were incorrect age (see above), small PHOMS dimensions and circumference (see above), missing PHOMS, inferior image quality ( < 25 dB on Spectralis Image Quality score, Q-value) and missing OCT scans. For ODD subjects, we excluded drusen significantly obscuring PHOMS measurements, simultaneous presence of ONH tilt, disc oedema and papilloedema. For IIH subjects, we excluded the simultaneous presence of a biomarker of pseudo-papilloedema, such as ONH tilt.

### Data acquisition

All participants underwent thorough examinations in the neuro-ophthalmology clinic at each participating site, where the anatomy of the ONH was examined using OCT according to the Optic Disc Drusen Studies Consortium guidelines [6]. The protocol included a 24-line radial OCT scan, a peripapillary 3-ring OCT scan, a dense ONH Enhanced Depth Imaging (EDI) OCT scan and a horizontal non-EDI macular OCT scan (Spectralis HRA + OCT; Heidelberg Engineering).

### Image analysis

One experienced grader in each site (CMB and JAF) assessed all included OCT scans. A PHOMS was identified as a hyperreflective ovoid mass-like structure on the peripapillary border of the ONH, forcing the retinal layers upwards in a ski-slope-like fashion [11]. The extent of PHOMS was evaluated using *en face* infrared fundus imaging or radial B-scans on ONH OCT. Only one eye of each patient was included. If bilateral PHOMS, the eye with the largest cross-sectional PHOMS area was used.

We defined the part of the retina directly above the PHOMS as the ‘PHOMS UP thickness’. For simplicity, ease of identification and reproducibility, we divided this layer into 2 distinct sublayers; first, the *hypo*reflective sublayer, extending from the outer nuclear layer (ONL) to the inner nuclear layer (INL); this sublayer is located directly on top of the PHOMS, secondly; the *hyper*reflective sublayer, consisting of the inner plexiform layer and attenuated ganglion cell layer (IPL-GCL) and the retinal nerve fibre layer (RNFL); this sublayer is limited anteriorly by the internal limiting membrane (ILM).

**Fig. 1:**
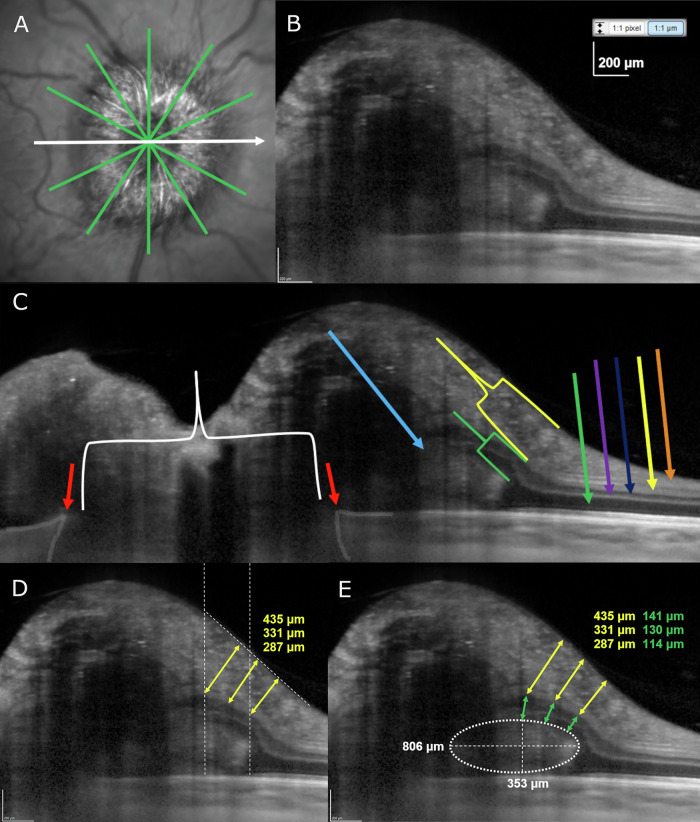
Key anatomical ONH structures and PHOMS UP measurement technique. En face infrared EDI-OCT of the right ONH of a patient with papilloedema due to IIH, showing the chosen midline section on the ONH, image **A** and corresponding cross-sectional midline OCT B-scan in correct 1:1 µm resolution, image (**B**). Image **C** shows key anatomical layers and structures of the ONH, from left to right: Bruch’s Membrane Opening (red arrows), ONH (white bracket) and PHOMS (blue arrow). The PHOMS UP layer is divided into two distinct sublayers: the hyporeflective sublayer (green bracket) above the PHOMS, consisting of the retinal layers ONL (green arrow), OPL (purple arrow) and INL (dark blue arrow) and the hyperreflective sublayer (yellow bracket), consisting of the IPL-GCL (yellow arrow) and RNFL (orange arrow). Image **D**, **E** step-by-step PHOMS UP measurement technique; The thickness of the hyperreflective layer is measured first as an average of three measurements perpendicular to the retinal layers and ILM. Firstly, the PHOMS is identified and two vertical lines are drawn through the top and lateral ends of the PHOMS. At the intersection of the vertical lines to the IPL-GCL, a line is drawn perpendicular to the ILM. The first measurement is made at the level of the top of the PHOMS, the second measurement at the level of the lateral end of PHOMS and the third in between the two former measurements (yellow arrows). Secondly, PHOMS dimensions are measured; cross-sectional height (white) is measured from the Bruch’s membrane vertically towards the top point of PHOMS. PHOMS cross sectional width (white) is measured horizontally from the lateral PHOMS end towards the medial PHOMS end at the ONH. Thirdly, the thickness of the hyporeflective sublayer is measured as an average of three measurements in continuation of the yellow arrows from the IPL-GCL down to the closest part of the PHOMS (green arrows). The green and yellow measurements together constitute the PHOMS UP thickness. This measurement sequence was then repeated at two additional peripapillary locations, each 30° from the central section. For each eye, the final PHOMS UP thickness value was calculated as the average of three measurements obtained in each of the three sections.

PHOMS height, width and PHOMS UP hypo- and hyperreflective sublayer thicknesses were measured at three peripapillary locations on OCT for each included eye. The first measurement was obtained at the B-scan showing the largest PHOMS cross-section (central PHOMS location), with the second and third measurements taken on 30° adjacent B-scans. At each location, PHOMS dimensions and sublayer thicknesses were recorded as illustrated in Fig. 1. If vessel shadowing obscured the PHOMS or sublayers, the next clockwise scan without shadowing was used. Final values were calculated as the average of the three measurements. The PHOMS delineation was evaluated based on the ease with which the medial margin of the PHOMS could be identified on OCT. OCT graders were not masked to the clinical diagnosis, as key features defining pseudo-papilloedema, including ODD and ONH tilt, were apparent on the OCT images.

Measurements were performed using the inbuilt HEYEX image analysis software on Heidelberg Spectralis (Heidelberg Engineering, Heidelberg, Germany). All images were analysed using the 1:1 µm resolution display to ensure accurate proportions for manual calliper placements.

### Statistics

RStudio software was used for statistical analysis. Normal distributions were evaluated by use of histograms for PHOMS dimensions and PHOMS UP thickness. Normally distributed data are presented as mean ± standard deviation (SD), while non-normally distributed data are presented as median with interquartile range (IQR). Student’s *t* tests were used to compare the pseudo-papilloedema group to the true papilloedema group. Chi-squared test was used for categorical data such as PHOMS medial delineation.

Univariable and multivariable logistic regression analysis were used to evaluate PHOMS dimensions and PHOMS UP thicknesses as predictors of true papilloedema. Results are presented as odds ratios (OR) with 95% confidence intervals. Sensitivity, specificity, positive predictive value (PPV) and negative predictive value (NPV) for the detection of true papilloedema were calculated on selected exploratory intervals of PHOMS UP thicknesses. Receiver operating characteristic (ROC) analysis was performed using continuous PHOMS UP thickness to determine the area under the curve (AUC) and the optimal cut-off using the Youden index. Spearman’s rank order (Spearman’s ρ) correlation test was used to assess the association between Frisén grades and PHOMS UP thickness. Intraobserver reproducibility was assessed and evaluated using Bland–Altman analysis.

The level of statistical significance was set at *P* < 0.05.

## Results

### Study population

In the pseudo-papilloedema group, OCT scans of 299 patients with either ODD or optic disc tilt were assessed. Exclusions were: 54 (age < 18), 123 (age > 45), 30 (missing ONH scans), 1 (poor image quality) and 11 (no PHOMS). Among the 80 remaining, 19 did not meet PHOMS size/extension criteria, 6 had PHOMS obscured by ODD, 5 had both ODD and ONH tilt, 2 with ODD had NA-AION and 1 had IIH, leaving 47 included patients. In the true papilloedema group, 48 IIH patients were assessed. Fourteen were excluded: 1 (age > 45), 3 (poor image quality), 1 (missing ONH scans), 1 (no PHOMS), 6 (not meeting PHOMS size/extension criteria) and 2 with concurrent pseudo-papilloedema from ONH tilt, yielding 34 included patients. Neither age (26.3 yrs ( ± 6.3) vs 28.9 ( ± 5.3), *P* = 0.051), sex (37 females and 10 males vs 31 females and 3 males, *P* = 0.230), nor laterality of the included eye (26 right and 21 left vs 18 right and 16 left, *P* = 1.000) differed between the pseudo-papilloedema and papilloedema group.

### Biomarkers of papilloedema

Intraobserver reproducibility of the PHOMS UP thickness and PHOMS height and width were assessed by repeat measurements performed four months apart by the same grader. The mean intraobserver difference was –0.8 µm ( ± 18.0 µm) for PHOMS UP thickness, –3.9 µm ( ± 47.3 µm) for PHOMS height and –28.4 µm ( ± 50.7 µm) for PHOMS width. Bland–Altman analysis showed no evidence of systematic or proportional bias. There were no statistical differences in PHOMS dimensions (cross-sectional height and width) between the pseudo-papilloedema group (*n* = 47) and the papilloedema group (*n* = 34), Table 1.

**Table 1:** OCT biomarkers.

	Pseudo-papilloedema	Papilloedema	*P* Value
**PHOMS DIMENSIONS**			
**Height, µm**	386.7 ( ± 76.4)	369.6 ( ± 59.3)	0.281*
**Width, µm**	502.5 ( ± 143.9)	562.5 ( ± 201.9)	0.122*
***PHOMS UP Layers***			
**1) Hyperreflective sublayer thickness, µm**	171.9 ( ± 39.4)	297.6 ( ± 54.2)	< 0.001*
**2) Hyporeflective sublayer thickness, µm**	31.7 ( ± 20.9)	80.7 ( ± 42.7)	< 0.001*
**1 + 2) Combined PHOMS UP thickness, µm**	203.6 ( ± 46.9)	378.3 ( ± 70.3)	< 0.001*
**OCT Signal Quality Score, Q-value, dB**	31.1 ( ± 3.7)	32.4 ( ± 4.2)	0.256
**PHOMS medial delineation,** ***Ω***			
**1 = good**	29	7	< **0.001¤**
**2 = moderate**	2	6	
**3 = poor**	2	15	

*PHOMS* peripapillary hyperreflective ovoid mass-like structure, *OCT* optical coherence tomography *Student *t* test ¤chi squared test *Ω*Only Copenhagen subjects.

The PHOMS UP thickness was significantly increased in the papilloedema group compared to the pseudo-papilloedema group for both sublayers: Hyperreflective sublayer thickness (297.6 µm ( ± 54.2 µm) vs 171.9 µm ( ± 39.4 µm), *P* < 0.001), hyporeflective sublayer thickness (80.7 µm ( ± 42.7 µm) vs 31.7 µm ( ± 20.9 µm), *P* < 0.001) and the combined PHOMS UP thickness (378.3 µm ( ± 70.3 µm) vs 203.6 µm ( ± 46.9 µm), *P* < 0.001), Table 1.

The PHOMS medial border delineation was significantly better in the pseudo-papilloedema group, where 29 of 33 had a well-defined medial border compared to 7 of 28 in the papilloedema group (*P* < 0.001), while Q-values were identical between the two groups (31.1 dB ( ± 3.7 dB) vs 32.4 dB ( ± 4.2 dB), *P* = 0.256), Table 1.

### Predictors of papilloedema

In a univariable logistic regression model, increased PHOMS dimensions (cross-sectional height and width) were not associated with increased odds of papilloedema, nor was age, sex or eye laterality. Increased PHOMS UP thickness was significantly associated with the presence of papilloedema in both a univariable and a multivariable model. In the univariable model, this was observed for the hyperreflective sublayer thickness (OR 3.77 (2.31–7.87), *P* < 0.001), hyporeflective sublayer thickness (OR 1.58 (1.32–2.00), *P* < 0.001) and the combined PHOMS UP thickness (OR 3.08 (1.99–6.19), *P* < 0.001, eTable 1. In the multivariable analysis adjusting for PHOMS dimensions (cross sectional width and height), age, sex and eye laterality, increased PHOMS UP thickness was still significantly associated with papilloedema, both in the hyperreflective sublayer thickness (OR 5.12 (2.68–14.77), *P* < 0.001), hyporeflective sublayer thickness (OR 1.63 (1.31–2.20), *P* < 0.001) and the combined PHOMS UP thickness (OR 4.23 (2.21–14.03), *P* = 0.001), eTable 1.

There was a positive and statistically significant correlation between the Frisén grade of the 34 participants with true papilloedema due to IIH and the PHOMS UP thickness, Spearman’s ρ 0.51, *P* = 0.002, Table 2.

**Table 2:** Spearman’s correlation between frisén grades and phoms up thickness.

Frisén Grades of IIH patients, *N* = 34	Distribution, *N* (%)	PHOMS UP thickness mean (SD) value, µm	PHOMS UP thickness median (IQR) value, µm	Spearman’s correlation coefficient (ρ)	*P* Value
**Grade 1**	3 (9%)	279 ( ± 35)	284 (263–298)		
**Grade 2**	12 (35%)	356 ( ± 78)	355 (286–417)		
**Grade 3**	15 (44%)	402 ( ± 40)	408 (364–426)		
**Grade 4**	4 (12%)	433 ( ± 73)	432 (377–488)		
**Grade 5**	0 (0%)			0.51	**0.002**

*IIH* idiopathic intracranial hypertension, *PHOMS* peripapillary hyperreflective ovoid mass-like structure, *SD* standard deviation, *IQR* interquartile range.

Examining the PHOMS UP thickness as a predictor of papilloedema, this quantitative biomarker was divided into appropriate cut-offs exploratively based on the observed means and distribution of measurements. A hyperreflective sublayer thickness of ≥200 µm was present in 94% with papilloedema vs 26% in the pseudo-papilloedema group, which resulted in a sensitivity of 94% and specificity of 74%. Raising the cut-off to ≥300 µm of the hyperreflective sublayer thickness, the sensitivity of detecting papilloedema decreased to 50%, but the specificity increased to 100%, Table 3.

**Table 3:** Diagnostic accuracy statistics and ROC analysis for detecting true papilloedema.

Predictors of True Papilloedema	Papilloedema vs. Pseudo-papilloedema	Sensitivity	Specificity	PPV	NPV
**PHOMS UP**					
**Hyperreflective sublayer thickness, µm**					
**≥200 µm**	32 vs 12	0.94	0.74	0.73	0.95
**≥250 µm**	26 vs 3	0.76	0.94	0.90	0.85
**≥300 µm**	17 vs 0	0.50	1.00	1.00	0.73
**PHOMS UP**					
**Hyporeflective sublayer thickness, µm**					
**≥50 µm**	25 vs 13	0.75	0.72	0.66	0.79
**≥100 µm**	14 vs 0	0.41	1.00	1.00	0.70
**Combined PHOMS UP thickness, µm**					
≥**300 µm**	28 vs 3	0.82	0.94	0.90	0.88

ROC Analysis	AUC (95% CI)	Sensitivity	Specificity	Optimal cut-off (µm)	Youden Index
**Combined PHOMS UP thickness, µm**	0.98 (0.96–1.0)	94%	89%	267	0.84

*ROC* Receiver Operating Characteristics, *PPV* positive predictive value, *NPV* negative predictive value, *PHOMS* peripapillary hyperreflective ovoid mass-like structure, *AUC* area under the curve.

A hyporeflective sublayer thickness of ≥50 µm was present in 75% of individuals with papilloedema and 28% in the pseudo-papilloedema group, which resulted in a sensitivity of 75% and a specificity of 72%. Increasing the cut-off to ≥100 µm, the sensitivity decreased to 41% while the specificity increased to 100%.

Combining these two layers, a PHOMS UP thickness of ≥300 µm was present in 82% of individuals in the papilloedema group and only in 6% of individuals in the pseudo-papilloedema group. Receiver operating characteristic (ROC) analysis of continuous PHOMS UP thickness yielded an area under the curve (AUC) of 0.98 (95% CI 0.96–1.00). The optimal threshold determined by the Youden index was 267 µm, corresponding to a sensitivity of 94% and a specificity of 89%, Table 3.

The biomarker with the highest PPV was the highest PHOMS UP cut-off value. The biomarker with the highest NPV was the hyperreflective sublayer thickness value of <200 µm, Table 3.

## Discussion

In this study, we examined a potential OCT biomarker, PHOMS UP thickness, to distinguish true papilloedema from pseudo-papilloedema in patients with co-existing and well-defined PHOMS based on ONH OCT of 81 patients with PHOMS due to either ODD, ONH tilt or IIH. We demonstrated that increased PHOMS UP thickness strongly suggests papilloedema when the hyporeflective sublayer thickness exceeds 50 µm, the hyperreflective sublayer thickness exceeds 250 µm, or the thickness of the combined layers, the PHOMS UP thickness, exceeds 267 µm. As we found 3 cases of ODD and 1 case of ONH tilt with PHOMS UP thickness around 300 µm, a threshold above 300 µm more reliably indicates true papilloedema, provided there are no co-existing ODD or significant ONH tilt.

Cross-sectional PHOMS size was similar between groups despite significant PHOMS UP thickness differences, suggesting the discriminative feature lies in the surrounding anatomy, not the PHOMS itself. We also found a moderately positive, statistically significant correlation between increasing Frisén grade and PHOMS UP thickness. This association may not be stronger because PHOMS UP thickness reflects localised thickening measured in selected radial ONH B-scans, while the Frisén grade reflects overall oedema.

Poor PHOMS medial delineation was more common in papilloedema despite comparable Q-values between the groups, likely reflecting the attenuating effect of light signal transduction on the deeper ONH structures by interstitial oedema.

### RNFL and PHOMS UP as biomarkers

We did not formally analyse ppRNFL thickness, as the primary objective was to evaluate PHOMS UP as a complementary structural parameter. Previous studies have examined ppRNFL thickness for differentiating papilloedema from pseudo-papilloedema, with Bassi et al. reporting moderate diagnostic performance for nasal ppRNFL (AUC 0.79) [12]. The ppRNFL thickness cannot reliably distinguish pseudo-papilloedema from true papilloedema, as ppRNFL may be increased in both conditions and subtle papilloedema may present with ppRNFL thickness within normal limits. Current OCT segmentation software does not identify or correct for PHOMS, instead measuring through ppRNFL at an angle rather than perpendicularly. In pseudo-papilloedema, interstitial oedema is minimal and elevation of the PHOMS UP layers (including ppRNFL) is primarily due to the PHOMS mass effect, whereas true papilloedema features both PHOMS-related displacement and actual thickening of the PHOMS UP layers due to interstitial oedema. This confounding effect highlights the need for a more anatomically grounded analysis. To keep our approach simple and clinically accessible, we excluded PHOMS and directly measured displaced tissue, assessing two predefined sublayers, both thickened in IIH. Multiple regression confirmed that even with fixed PHOMS dimensions, both PHOMS UP sublayer thicknesses remained significantly increased in true papilloedema. The PHOMS UP measurement encompasses all retinal layers located above the PHOMS and therefore reflects inner retinal thickness directly at the ONH. Although it may partially overlap conceptually with ppRNFL or total retinal thickness, PHOMS UP differs in that it interrogates tissue at the epicentre of maximal architectural distortion and interstitial oedema. In contrast, conventional peripapillary ring measurements are obtained at a fixed radial distance from the disc centre, where oedema may be less pronounced and structural displacement due to PHOMS may not be fully captured.

PHOMS UP thickness is essentially the Bruch’s Membrane Opening–minimum rim width (BMO-MRW) minus PHOMS. BMO-MRW, the minimum distance from Bruch’s membrane to the ILM, is an OCT biomarker mainly used in glaucoma. Our results align with the only prior study comparing BMO-MRW in papilloedema and pseudo-papilloedema, where Swanson et al. reported higher values in papilloedema (median 576.7 μm vs. 478.3 μm) [13]. Unlike BMO-MRW, which also reflects PHOMS mass effect, PHOMS UP isolates true tissue thickening from mechanical displacement.

**Fig. 2:**
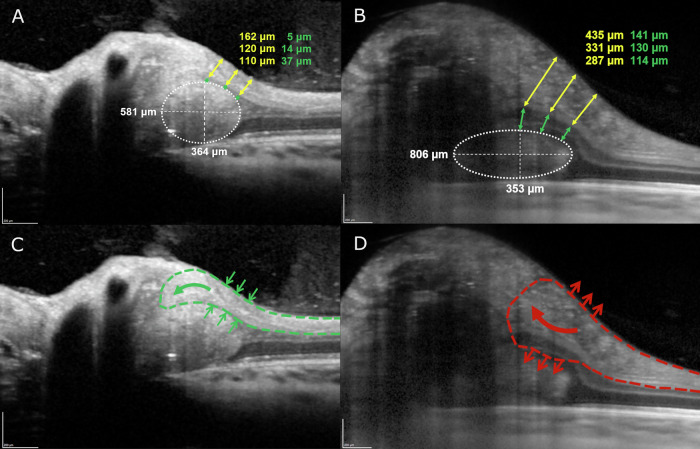
Key PHOMS UP differences and Cobra sign. **A** pseudo-papilloedema due to ODD and **B** true papilloedema due to IIH. PHOMS are of equal heights on cross section, hyporeflective sublayer thickness is < 50 µm in pseudo-papilloedema compared to > 100 µm in true papilloedema, hyperreflective sublayer thickness is < 200 µm in pseudo-papilloedema (here, even < 150 µm) vs > 300 µm in true papilloedema. **C**, **D**: To conceptualise the clear distinction between the PHOMS UP thicknesses in a qualitative manner, we introduce the “cobra sign”: in pseudo-papilloedema, the PHOMS UP layer resembles a harmless garter snake (in green), slim and low-lying. In true papilloedema, it resembles a cobra (in red) in an aggressive stance, with its thickened neck part—called the hood—raised when threatened.

Figure 2 illustrates the difference in PHOMS UP thickness and configuration, with sublayers remaining relatively unaltered in pseudo-papilloedema but thickened in true papilloedema.

### PHOMS

In a large cohort of patients with ODD, 77% of eyes had PHOMS, with higher frequency in younger individuals and declining prevalence with age [14], while in a series of patients with IIH, 81% of eyes had PHOMS [15]. Furthermore, in the Copenhagen Child Cohort 2000, a PHOMS was identified in approximately 9% of otherwise healthy 10–11-year-old children without ODD or papilloedema, predominantly in association with myopia and tilted ONHs. Some researchers theorise that certain PHOMS sizes, shapes or morphologies might be specific to the underlying disease causing the PHOMS [1, 16]. One might therefore think that a PHOMS differs in size between true papilloedema and pseudo-papilloedema. In this study, we evaluated the cross-sectional size of PHOMS (measured as height and width) as a surrogate marker of volume, disregarding total extent and morphology. PHOMS typically follow the NSIT rule of distribution (nasal > superior > inferior > temporal) [3, 14] and are more generalised in true papilloedema compared to pseudo-papilloedema due to ONH tilt [1, 3, 5]. To account for PHOMS size, we only included patients with PHOMS covering ≥25% of the peripapillary circumference and measuring ≥300 µm on central and ≥150 µm on lateral cross sections. Inclusion of smaller PHOMS would have introduced substantial variability in PHOMS dimensions within and between groups, potentially obscuring differences attributable to mass effect. As we did not define an upper limit for PHOMS size, we were surprised to find that cross-sectional PHOMS size did not differ significantly between groups (Table 1). Height was consistent, while width was smaller in pseudo-papilloedema but not significantly so. This may reflect either a more elongated configuration in papilloedema or, more likely, difficulty defining the medial PHOMS border due to interstitial oedema and OCT shadowing in papilloedema. Supporting the latter explanation, medial borders were significantly easier to delineate in pseudo-papilloedema (Table 1). One can argue that an ill-defined medial border may itself represent a marker of interstitial oedema and thus papilloedema.

Overall, total PHOMS volume was certainly higher in true papilloedema, but this reflects circumferential distribution rather than radial section size.

### Implications for pseudo-papilloedema with superimposed true papilloedema

A relevant clinical dilemma arises when pseudo-papilloedema patients (e.g., with ODD-associated PHOMS) develop true disc oedema, such as ODD-AION or IIH. Given an ODD prevalence of ~2% [17], this scenario is not rare. In such cases, PHOMS UP thickness may prove valuable, as ODD with superimposed disc oedema would be expected to show increased PHOMS UP thickness. This is yet to be investigated.

### Strengths and limitations

Key strengths include the large sample size, double-centre design and involvement of two experienced neuro-ophthalmologists (SH, JAF) and an expert OCT grader (CMB). Several limitations should be acknowledged. Importantly, this study represents a highly selected cohort, as inclusion was restricted to patients with well-defined and sufficiently extensive PHOMS in exclusively young adults. These criteria were predefined to enable assessment of PHOMS-related mass effect on the overlying retinal layers, but resulted in the exclusion of a substantial proportion of screened patients. Notably, in the pseudo-papilloedema cohort, many excluded patients were screened out prior to OCT assessment, primarily due to age outside the predefined range of 18–45 years. In addition, the predefined PHOMS size criteria required medium to large PHOMS, thereby excluding small PHOMS and potentially underrepresenting early or mild papilloedema. Indeed, only a small proportion of included papilloedema cases were Frisén grade 1. Consequently, the findings may not be generalisable to patients with small PHOMS, subtle or early papilloedema, mixed phenotypes (such as coexisting optic disc tilt and papilloedema) and are not applicable to eyes without PHOMS. Furthermore, the true papilloedema cohort consisted exclusively of patients with IIH. Whether PHOMS UP measurements perform similarly in papilloedema caused by other aetiologies, such as intracranial tumours or other causes of raised intracranial pressure, remains to be determined. Interobserver variability was not formally assessed, as OCT images could not be transferred between sites. However, all measurements were performed according to a predefined standardised protocol and the low SDs relative to means (Table 1) indicate low variability, consistent with the high specificity of PHOMS UP thickness (Table 3). In addition, intraobserver reproducibility was assessed by repeat measurements performed by an experienced grader (CMB) four months after the initial assessment, demonstrating good measurement consistency with no evidence of systematic or proportional bias, supporting the robustness of the PHOMS UP measurement approach in the hands of a trained observer. Finally, eyes with severe papilloedema (Frisén grade 5) were excluded because extensive oedema obscured retinal layer boundaries and precluded reliable OCT-based measurements despite high Q-values. The applicability of PHOMS UP thickness in advanced papilloedema, therefore, remains uncertain.

### Conclusions and future perspectives

In this study, we evaluated the thickness of the retinal layers above PHOMS as a quantifiable OCT-derived parameter for distinguishing true papilloedema from pseudo-papilloedema in patients with well-defined PHOMS. The method is simple, widely applicable and provides complementary information to existing methods, such as ppRNFL assessment alone. In patients with suspected optic disc oedema from raised ICP, a PHOMS UP thickness exceeding 300 µm points in the direction of true papilloedema and may reduce its overdiagnosis.

## Summary

### What is known about this topic

- Differentiating pseudo-papilloedema from true papilloedema is crucial as the latter can be both vision and life-threatening.
- Optic Coherence Tomography (OCT) can visualise structural changes of the optic nerve head (ONH) but lacks the ability to differentiate between the two conditions.
- Peripapillary Hyperreflective Ovoid Mass-like Structures (PHOMS) are novel findings on OCT found in both pseudo- and true papilloedema, limiting their diagnostic specificity.

### What this study adds

- Introduces a new, rapid OCT-derived metric, the PHOMS UP Thickness, to aid in distinguishing true papilloedema from pseudo-papilloedema in patients with well-defined PHOMS.
- Demonstrates that a PHOMS UP thickness of more than 300 microns is suggestive of true papilloedema, reflecting that true papilloedema involves both intra-axonal and interstitial fluid stasis.

## Supplementary information

eTABLE 1

Eye Research Checklist

## Data Availability

The data that support the findings of this study are available from the Department of Ophthalmology, Copenhagen University Hospital, Copenhagen, Denmark and the Department of Ophthalmology and Department of Clinical Neurology, Western University, London, Ontario, Canada, but restrictions apply to the availability of these data, which were used under license for the current study and are therefore not publicly available. Data are, however, available from the authors upon reasonable request and with permission of the data-owning institutions.
