# Supplementary material for: Optical coherence tomography measurement of retinal layers above the peripapillary hyperreflective ovoid mass-like structure in true papilloedema and pseudo-papilloedema
Source: Eye (Lond). 2026 May 21;40(11):1720–7. doi: 10.1038/s41433-026-04522-0 (PMC13416131; doi:10.1038/s41433-026-04522-0)
Supplement: Supplementary file 1 — eTABLE 1 [file 41433_2026_4522_MOESM1_ESM.docx]

**eTABLE 1**: UNIVARIABLE AND MULTIVARIABLE LOGISTIC MODELS OF PREDICTORS OF PAPILLOEDEMA

|  | Univariable model | | | Multivariable model¤ | | |
| --- | --- | --- | --- | --- | --- | --- |
|  | OR | 95% CI | P Value | OR | 95% CI | P Value |
| PHOMS DIMENSIONS Height, µm Width, µm  *Per 50 µm increase* | 0.81 1.12 | (0.53-1.15) (0.97-1.31) | 0.291 0.142 |  |  |  |
| PHOMS UP Hyperreflective sublayer thickness, µm *Per 25 µm increase* | 3.77 | (2.31-7.87) | **< 0.001** | 5.12 | (2.68-14.77) | **< 0.001** |
| PHOMS UP Hyporeflective sublayer thickness, µm *Per 10 µm increase* | 1.58 | (1.32-2.00) | **< 0.001** | 1.63 | (1.31-2.20) | **< 0.001** |
| Combined PHOMS UP thickness, µm  *Per 25 µm increase* | 3.08 | (1.99-6.19) | **< 0.001** | 4.23 | (2.21-14.03) | **0.001** |
| Age, yrs | 1.08 | (1.00-1.17) | 0.061 |  |  |  |
| Sex (Female vs Male) | 0.36 | (0.08-1.29) | 0.143 |  |  |  |
| Eye (Right vs Left) | 1.10 | (0.45-2.68) | 0.832 |  |  |  |

¤Adjusted for PHOMS height, PHOMS width, age, sex and eye side.
OR = odds ratio; CI = confidence interval; PHOMS = peripapillary hyperreflective ovoid mass-like structure
